# Acceptability of policies to reduce consumption of red and processed meat: A population-based survey experiment

**DOI:** 10.1016/j.jenvp.2022.101817

**Published:** 2022-06

**Authors:** Rachel Pechey, James P. Reynolds, Brian Cook, Theresa M. Marteau, Susan A. Jebb

**Affiliations:** a University of Oxford, Radcliffe Primary Care Building, Radcliffe Observatory Quarter, Woodstock Road, Oxford, OX2 6GG, UK; b University of Cambridge, Cambridge Institute of Public Health, Forvie Site, Cambridge, CB2 0SR, UK; c School of Psychology, Aston University, Birmingham, B4 7ET, UK

**Keywords:** Meat, Acceptability, Policy, Framing, Environment, Health

## Abstract

Policies to reduce meat consumption are needed to help achieve climate change targets, and could also improve population health. Public acceptability can affect the likelihood of policy implementation. This study estimated the acceptability of policies to reduce red and processed meat consumption, and whether acceptability differed when policies were framed as benefitting health or the environment. In an online experiment, 2215 UK adults rated the acceptability of six policies, presented in a randomised order. Prior to rating policies, participants were randomised to one of two framing conditions, with policy outcomes described either as benefitting health or the environment. Regression models examined differences in the primary outcome – policy acceptability (rated on a 7-point scale) – by framing. Labels were the most accepted policy (48% support), followed by a media campaign (45%), reduced availability (40%) and providing incentives (38%). Increasing price (27%) and banning advertising (26%) were the least accepted. A substantial proportion of participants neither supported nor opposed most policies (26–33%), although this fell to 16% for increasing price. There was no evidence that framing policy benefits from a health or environment perspective influenced acceptability (−0.06, 95%CIs: 0.18,0.07). Fewer than half of the UK sample expressed support for any of six policies to reduce meat consumption, regardless of framing measures as benefitting health or the environment. Conversely, fewer than half expressed opposition, with the exception of price, suggesting considerable scope to influence public opinion in support of meat reduction measures to meet environmental and health goals.

## Highlights

• Fewer than half this UK-representative sample supported meat reduction policies.
• Framing measures as benefitting health vs. the environment did not change support.
• Policies targeting meat were less supported than policies targeting unhealthy food.
• Many respondents had no decided views about the acceptability of policies on meat.
• There may be considerable scope to increase support for meat reduction policies.

## 1 Background

The UK Climate Change Committee suggests that a reduction in consumption of beef, lamb and dairy of at least 20% per person by 2030 is required to deliver the UK's target of net zero carbon emissions (Climate Change Committee, 2020). While meat consumption in the UK is decreasing, dietary change will need to accelerate to meet sustainability targets (Stewart, Piernas, Cook, & Jebb, 2021). Given that 56% of men and 24% of women exceeded the UK Scientific Advisory Committee on Nutrition's recommended maximum daily red and processed meat intake in 2016/17 (Stewart, Piernas, Cook, & Jebb, under review), reducing red and processed meat consumption could lead to substantial benefits both in terms of health and environmental impacts (Springmann et al., 2018). The scale of change needed is likely to require specific policy action, and the likelihood of policies being introduced depends – in part – on public acceptability (Cullerton et al., 2016, Cullerton et al., 2018).

A systematic review of attitudes to environmental concerns regarding meat consumption found few studies that looked at the acceptability of specific meat reduction policies (Sanchez-Sabate & Sabaté, 2019) thus limiting the conclusions that can be drawn from the existing literature. Studies have often focused on a narrow context, e.g., surveying students about policies to be implemented in university cafeterias (De Groeve & Bleys, 2017), or only considered a limited range of policy options (predominantly focused on taxes or subsidies) (Graça, Cardoso, Augusto, & Nunes, 2020). However, previous research in other areas of public health policy has highlighted that public support tends to be stronger for non-fiscal policies – and in particular those that involve provision of information, with greater support for educational campaigns than for taxation or restrictions on sales (Diepeveen, Ling, Suhrcke, Roland, & Marteau, 2013). More intrusive yet less popular policies can however often be more effective in terms of behaviour change (Marteau et al., 2019).

Greater awareness of the harms associated with a behaviour, such as meat consumption, may increase support for policies restricting that behaviour (Diepeveen et al., 2013). Given that public understanding of the links between diet and health is generally high (de Ridder, Kroese, Evers, Adriaanse, & Gillebaart, 2017; Lappalainen, Kearney, & Gibney, 1998; Paquette, 2005), whereas the impact of food choices on sustainability is less well established (Macdiarmid, Douglas, & Campbell, 2016; Sanchez-Sabate & Sabaté, 2019), framing meat reduction policies in terms of their impact on health (vs. impact on the environment) may serve to increase public acceptability for these policies.

This study aimed to estimate the acceptability of policies to reduce red and processed meat consumption, and to assess whether acceptability differed when policies were framed as benefitting health or the environment. The study also aimed to examine whether acceptability varied with participant demographics, or attitudes and behaviours related to meat consumption.

## 2 Methods

The study was pre-registered on the Open Science Framework (https://osf.io/pxhvr). The project was reviewed by, and received ethics approval, through the University of Oxford Central University Research Ethics Committee [R71792/RE001]. All participants provided consent to participate.

Design: In a between-subjects design, participants completing the online experiment were randomised (via Qualtrics) to one of two groups varying by the study introduction that described policies as having either health or environmental benefits. Each participant saw six policies presented in a random order.

Participants: A sample of 2252 participants was recruited from a market research agency panel (Dynata; www.dynata.com), with quotas set to obtain a UK-representative sample by age, gender and education. Speeders (those completing the survey in less than 30% of the median completion time) were excluded (n = 37), leaving a final sample of 2215 UK adults.

A sample size of 2250 was calculated to provide 90% power to detect a 0.25 unit change on the 7-point scale used to assess acceptability (selected as the smallest change of interest; an effect size of 0.0166; with S.D. of 1.5, based on values seen in similar studies (Mantzari et al., 2022: pilot data)) between health vs. environmental framing, for a 2-tailed *t*-test, with alpha set to 0.008 to adjust for each of the six policies to be examined (calculated using G*Power 3.1.9.2).

Intervention: Participants were randomised to one of two introductory statements that highlighted either (a) health or (b) environmental benefits of reducing meat consumption. In both study conditions, this introduction began with the statement “The government is considering a number of policy options to reduce the amount of red and processed meat that people eat”. For the health condition the following sentence read: “This will improve people's health by reducing the number of cases of colorectal cancer and heart disease” and for the environment this was replaced with “This will help protect the environment by reducing greenhouse gas emissions and water pollution”. At the end of the introduction, a definition of red and processed meat was included in both study conditions (“Red meat includes beef, lamb and pork. Processed meat includes meat that's been altered by being cured, salted, or smoked (such as bacon, sausages, and ham)”).

### 2.1 Materials and measures

Six examples of meat reduction policies were assessed, selected to represent a range of levels of intrusion on the Nuffield intervention ladder (Nuffield Council on Bioethics, 2007):

1. Media campaign: “A government media campaign that highlights the harms of eating red and processed meat.”
2. Incentives for producers: “Providing government grants to beef producers who switch to farming pulses and legumes.”
3. Labels: “A policy requiring labels on all products containing red or processed meat that highlight the harms of red and processed meat.”
4. Reduced availability: “Limits on the proportion of meals containing red or processed meat at hospitals and other public sector organisations. This would mean no more that 25% of meals would contain red or processed meat.”
5. Increased price: “A tax that raised the price of red and processed meat by 20%. This would mean, for example, that a 500g pack of mince that currently costs £2.50 would instead cost £3.00.”
6. Advertising ban: “Banning all advertising of products containing red or processed meat.”

#### 2.1.1 Measures

##### 2.1.1.1 Acceptability

The question format was based on that used in previous studies (Petrescu, Hollands, Couturier, Ng, & Marteau, 2016; Reynolds, Pilling, & Marteau, 2018), with responses measured on a 7-point response scale, labelled from “Strongly oppose” to “Strongly support”.

##### 2.1.1.2 Behaviour - meat consumption

Participants were asked to self-report their usual meat consumption. Consumption scores were the sum of two questions asking how often participants usually eat [any kind of meat (including chicken, fish and seafood)][red or processed meat] as part of (1) their dinner/tea, or (2) their lunch. The two original questions were scored 0 (“Never”); 1 (“Less than once a week”); 2 (”1–2 times a week”); 3 (”3–4 times a week”); 4 (”5–6 times a week”); 5 (“Every day”).

Participants also reported the likelihood of them reducing their meat consumption within the next six months (from 0 “Extremely unlikely” to 6 “Extremely likely”), both overall and specifically for red and processed meat.

##### 2.1.1.3 Beliefs and attitudes

Importance of health/the environment: Two single-item variables in which participants were asked to rate the importance of “Everybody in the population having good health, including not getting cancer” and “Everything being done to protect the environment, including preventing climate change” (using scales from 0 = not at all important; 6 = very important (Mantzari et al., 2022)).

Impact of meat consumption on health/the environment (Tobler, Visschers, & Siegrist, 2011): Two single-item variables in which participants rated whether they agreed or disagreed that “Reducing red and processed meat consumption is healthier” and “Reducing red and processed meat consumption is better for the environment” (using scales from 0 = completely disagree; 6 = completely agree).

Trust in the UK government: A single item developed for this study, participants were asked how far they tended to trust the UK government, using ratings from 0 = Distrust it greatly; 6 = Trust it greatly.

### 2.2 Procedure

The study was conducted online via Qualtrics (an online survey platform). Participants first completed questions relating to age, gender and education (relating to study quotas).

Participants were then randomised to see one of the two introductory statements that highlighted either (a) health or (b) environmental benefits of reducing meat consumption.

Following their allocated introduction, participants were asked whether they would support or oppose each of the six policies, presented in a random order.

Participants then completed questions regarding their ethnicity, annual household income and home postcode (used to obtain the Index of Multiple Deprivation), as well as responding to questions on meat consumption and the measures of beliefs and attitudes.

### 2.3 Analysis

Multilevel linear regression models (accounting for repeated measures from each participant) were used to assess policy acceptability ratings, with predictors being dummy variables for policy type and whether the introduction highlighted health or the environment.

Differences between benefits being presented in terms of health vs. sustainability were tested for each policy, using t-tests. A significant effect was inferred if any p-value was lower than α = 0.008; following a Bonferroni adjustment to account for six policies.

Multiple regression analyses modelled the mean acceptability score, from predictor variables: gender, age, socioeconomic status, ethnicity, typical [red and processed] meat consumption, intentions to reduce [red and processed] meat consumption, perceived importance of health and sustainability, belief in the impact of meat consumption on health and the environment, and trust in government. An alpha value of 0.003 (using a Bonferroni adjustment to take account of the 15 different predictors) was used for these analyses.

## 3 Results

Of the 2215 completers, 48% were male and 52% female; with an age range of 18–98 (mean 47.3; s.d. 17.1). Table 1 shows that 53% fell into the higher education group (2 or more A-levels or equivalent, and above), with 87% of participants who reported ethnicity being white.

**Table 1:** Participant characteristics, beliefs and attitudes towards meat, by condition.

		Environment	Health	Total
*n*		1106	1109	2215
% (n)				
Age group	18–24	11.2 (124)	11.8 (131)	11.5 (255)
	25–34	17.4 (192)	16.4 (182)	16.9 (374)
	35–44	17.2 (190)	18.8 (208)	18.0 (398)
	45–54	18.2 (201)	17.6 (195)	17.8 (396)
	55–64	15.4 (170)	15.2 (169)	15.3 (339)
	65+	20.7 (229)	20.2 (224)	20.5 (453)
Gender	Male	47.0 (519)	49.5 (549)	48.3 (1068)
	Female	52.8 (583)	50.3 (557)	51.5 (1140)
	Other	0.03 (3)	0.02 (2)	0.02 (5)
Education	Higher	46.0 (509)	49.0 (543)	47.5 (1052)
	Lower	54.0 (597)	51.0 (566)	52.5 (1163)
Ethnicity	White	87.4 (958)	87.4 (963)	87.4 (1921)
	Other	12.6 (138)	12.6 (139)	12.6 (277)
Mean (s.d.)				
Meat (any) consumption		5.25 (2.29)	5.24 (2.33)	5.24 (2.31)
Meat (red) consumption		3.67 (2.22)	3.68 (2.30)	3.68 (2.26)
Intend to reduce meat consumption (any meat)		2.43 (1.71)	2.39 (1.70)	2.41 (1.71)
Intend to reduce meat consumption (red meat)		2.71 (1.80)	2.60 (1.80)	2.65 (1.80)
Importance of population health		4.50 (1.45)	4.48 (1.40)	4.49 (1.43)
Importance of protecting the environment		4.09 (1.60)	4.09 (1.58)	4.09 (1.59)
Belief that eating less meat is good for health		3.74 (1.62)	3.66 (1.64)	3.70 (1.63)
Belief that eating less meat is good for environment		3.66 (1.65)	3.58 (1.69)	3.62 (1.67)
Trust in government		2.73 (1.61)	2.67 (1.61)	2.70 (1.61)

N.B. Percentages are of non-missing values: 2 participants did not report gender (one in each condition); 17 participants did not report ethnicity (10 in environment condition; 7 in health condition); participants who reported never eating meat were not asked the intentions to reduce meat questions (n = 103; 47 in the environment condition and 56 in the health condition).

**Fig. 1:**
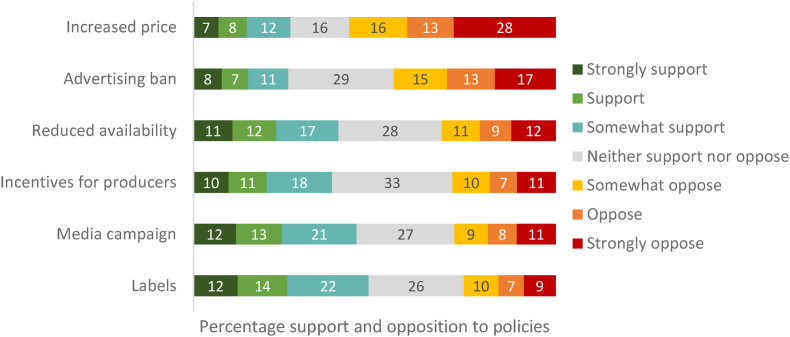
Policy support vs. opposition (combined over health and environment conditions). NB: Due to rounding, the sum of these values may not match the percentages in the text.

Four policies received more support than opposition. Labels were the most acceptable policy with 48% (n = 1072) indicating they supported this to some extent, vs. 25% (n = 562) opposing to some extent, followed by a media campaign (45% (n = 997) support vs. 28% (n = 620) oppose) (see Fig. 1). Reduced availability of meat options (40% (n = 887) support vs. 31% (n = 697) oppose) and providing incentives for producers to switch to more sustainable crops (38% (n = 849) support vs. 29% (n = 632) oppose) were also more likely to be supported than not. In contrast, more people opposed than supported the advertising ban (26% (n = 580) support vs. 45% (n = 989) oppose) and increased price (27% (n = 594) support vs. 57% (n = 1262) oppose). All other policies were significantly more acceptable than increasing the price of meat (increases of between 0.4 and 1.1 in mean policy acceptability scores; see Supplementary Table S1). Between 16 and 33% of the sample neither supported or opposed these policies.

**Fig. 2:**
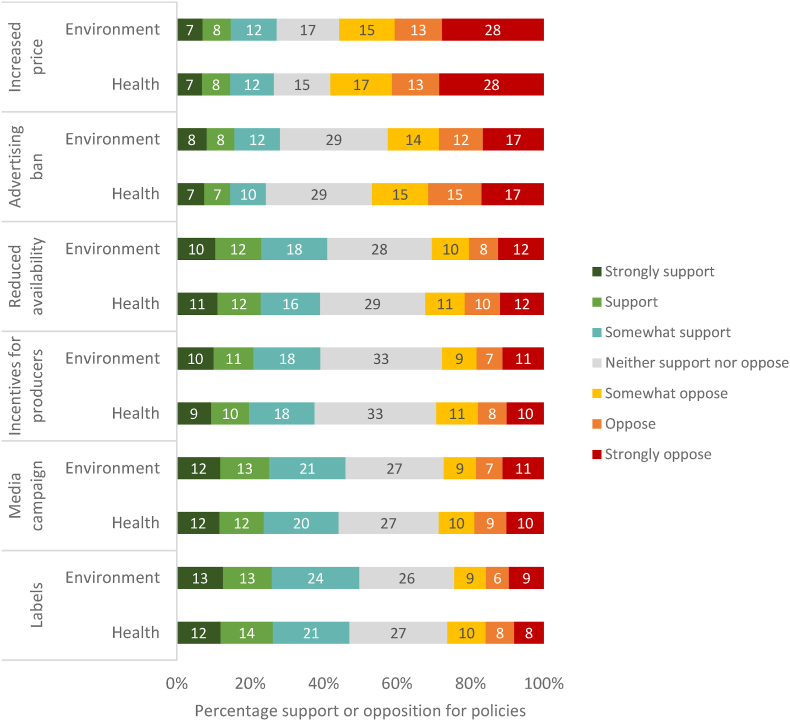
Policy support vs. opposition by condition.

There was no evidence of a difference in the overall acceptability of policies when introduced as targeting health or environmental concerns (Supplementary Table S1: regression coefficient for health frame (relative to environment frame): −0.06; 95%CIs: −0.18, 0.07), nor any evidence of a difference in the acceptability for any individual policy between these frames (Fig. 2; Supplementary Table S1).

Differences by participant demographics, behaviour or attitudes: Given that there was no evidence of a difference between the health and environment conditions, the planned separate analyses for health and environment conditions (https://osf.io/pxhvr) were combined (see Supplementary Table S2 for sensitivity analyses showing these). No evidence was found to suggest age, gender, education or ethnicity were associated with policy acceptability (see Table 2).

**Table 2:** Regression analyses modelling mean policy acceptability by participant characteristics.

		Coefficient	95% CIs	p-value
Age		−0.003	−0.006, −0.003	0.028
Gender (ref: Male)	Female	−0.02	−0.10, 0.07	0.685
	Other	−0.45	−1.37, 0.47	0.339
Education (ref: Lower)	Higher	0.03	−0.05, 0.12	0.455
Ethnicity (ref: Other)	White	0.02	−0.11, 0.15	0.750
Meat (any) consumption		**−0.05**	**−0.07, -0.02**	**<0.001**
Meat (red) consumption		−0.04	−0.06, −0.01	0.005
Intend to reduce meat consumption (any meat)		**0.09**	**0.04, 0.13**	**<0.001**
Intend to reduce meat consumption (red meat)		**0.16**	**0.12, 0.20**	**<0.001**
Importance of population health		−0.0009	−0.04, 0.04	0.963
Importance of protecting the environment		**0.11**	**0.08, 0.15**	**<0.001**
Belief that eating less meat is good for health		**0.16**	**0. 11, 0.20**	**<0.001**
Belief that eating less meat is good for environment		**0.22**	**0.18, 0.26**	**<0.001**
Trust in government		**0.06**	**0.04, 0.09**	**<0.001**
Constant		0.75	0.49, 1.01	<0.001

A p-value of <0.003 indicates significance.

N.B. 2097 observations (17 participants did not report ethnicity, 2 did not report gender (1 of whom did not report either ethnicity or gender), 103 did not answer the intentions to reduce meat question (of whom 3 did not report ethnicity)).

Higher total meat consumption was associated with lower acceptability ratings. Stronger intentions to reduce red and processed meat, or overall meat, consumption were associated with higher policy acceptability.

Higher importance ratings for protecting the environment, but not for protecting population health, was associated with policy acceptability. Stronger beliefs that eating less meat is good for health and that eating less meat is good for the environment were also associated with higher policy acceptability, as was greater trust in the government.

In sensitivity analyses where the intentions to reduce meat questions were removed from analyses (as these were missing for non-meat-eaters), results were largely similar (see Supplementary Table S3), however, the relationship between age and acceptability became statistically significant (coefficient: −0.007; 95% CIs: −0.010, −0.004; p < 0.001).

## 4 Discussion

In a large sample representative of the UK population there was between 27 and 48% support for – and 25–57% opposition to – policies to reduce meat consumption, which did not differ if framed as improving health or enhancing environmental sustainability. Support and opposition was also unrelated to age, gender, education or ethnicity. Policies that were information-based (e.g., media campaigns) had greater public support than policies targeting industry actions or imposing financial penalties. Several variables were associated with policy support: greater importance being placed on protecting the environment, belief that eating less meat is good for health or the environment, and personal intentions to eat less meat all were associated with higher acceptability of policy action.

These results largely support and build on previous research on acceptability of other policy topics (Diepeveen et al., 2013; Hagmann, Siegrist, & Hartmann, 2018; Petrescu et al., 2016), with policies providing information (labelling and media campaign) being most acceptable. In contrast, more people opposed than supported the advertising ban or an increase in the price of meat. However, information-based policies are more likely to rely on an individual's cognitive resources to be successful, which may limit their effectiveness, as well as potentially increasing inequalities (Adams, Mytton, White, & Monsivais, 2016; Marteau, Hollands, & Fletcher, 2012; Rutter et al., 2017). In contrast, the less popular policies – price, advertising and store environment practices – may be the more effective (Marteau et al., 2019).

This study explored whether framing policies as targeting health rather than environmental concerns might increase acceptability. However, there was no evidence of any differences depending on the framing used – consistent with results from a recent study suggesting that health and environmental framing both worked better than economic or migration frames, but finding no difference between the two (Dasandi et al., 2021). Indeed, in contrast to the hypothesised greater awareness for the impact of meat consumption on health, levels of belief in eating less meat being good for health vs. good for the environment showed only a small difference (mean ratings of 3.7 vs. 3.6), possibly negating any potential advantage from the health framing. This may reflect increasing awareness of the contribution of meat consumption to environmental impact in the population (YouGov, 2019). It is also possible that the framing intervention used – which represented a more subtle and low-engagement frame (Petrovic, Madrigano, & Zaval, 2014), rather than e.g. participants reading specially crafted news articles (Myers, Nisbet, Maibach, & Leiserowitz, 2012) – was not salient enough to produce differences. That said, in a previous study when participants were asked to actively contrast different statements, health and environment frames did not differ (Dasandi et al., 2021). In addition, one US study using a similar method of framing suggested that public support was moderated by political orientation – with a climate frame producing stronger support for liberals and a health frame for conservatives when rating support for options that required external intervention, such as government policies (Petrovic et al., 2014). The authors interpret this in terms of identity affirmation, with individuals dismissing information that opposes their values (Kahan, 2010). Further exploration of participants’ willingness to support government intervention in general, along with exploring alternative framing approaches, would help to establish whether framing could impact on support for meat reduction policies.

We found several variables that correlated with public acceptability. Intending to reduce meat consumption, placing greater importance on protecting the environment, and beliefs that eating less meat is good for health and for the environment, predicted policy acceptability regardless of study condition. These factors are again largely consistent with findings from previous research, with those who engage less in the targeted behaviour, or who are more aware of the harms associated with a behaviour or of evidence of policy effectiveness being more likely to find policies acceptable (Diepeveen et al., 2013; Holmila, Mustonen, Österberg, & Raitasalo, 2009; Wilkinson, Room, & Livingston, 2009). This implies that if the trends towards greater environmental concern (Ipsos-MORI, 2019) or eating less meat continue (Stewart et al., 2021), the acceptability of policy action may increase, though the rate of change is currently too slow to meet policy goals. Moreover, as one recent poll noted (YouGov, 2018), despite increasing support for the idea of eating less red meat, as yet there seems little appetite for government intervention to achieve this – in contrast to policies targeting healthy diets. As a first step, research is needed on how public support for effective strategies to meat reduction can be boosted. Our study findings suggested trust in the government shaped policy acceptability, and other stakeholders with higher trust ratings or credibility with the public may be needed to boost trust and increase the acceptance of meat reduction policies (Huang et al., 2015). In addition, a recent meta-analysis suggested that perceived fairness and effectiveness were key factors in determining acceptability of policies targeting climate change (Bergquist, Nilsson, Harring, & Jagers, 2022).

The Climate Change Committee (2020) has suggested a 20% reduction in meat and dairy consumption by 2030 will be necessary for government to achieve its net zero target. Some reports have suggested reductions of up to 85% will be required to meet broader environmental goals, including water use, biodiversity, land use and nitrogen and phosphorus application (Willett et al., 2019). Fewer than half those sampled expressed support for even the least intrusive policies, however. Support was lower than in a previous study where all policies aimed at reducing the consumption of alcohol, tobacco and snack foods were supported by the majority of participants (Reynolds et al., 2019). This ties in with a recent study including both high energy dense foods and meat as reduction targets, which suggested policy acceptability of 48% vs. 27% respectively (Mantzari et al., 2022) – highlighting the major challenge of getting the public to support meat reduction initiatives. There are strong social and cultural norms with regard to meat eating across many countries with high levels of consumption (Modlinska & Pisula, 2018), in which meat consumption is regarded as ‘necessary, natural, normal and nice’ (Mullee et al., 2017; Piazza et al., 2015). Indeed, targeting meat reduction is generally more unpopular than other climate change strategies, with one study suggesting this to be the case even amongst those who were most concerned about climate change (Maibach, Nisbet, Baldwin, Akerlof, & Diao, 2010). That said, while public acceptability can play a role in decisions regarding policy implementation, it is only one of several factors that may be considered (Moberg et al., 2018). It is possible in some cases that other pressing factors may counteract relatively low acceptability, removing this as an obstacle to policy action, though low acceptability can also impede subsequent implementation.

However, a substantial proportion of participants, between 26 and 33%, neither supported nor opposed the suggested meat reduction policies, with the exception of increasing price (16%) where most people had clear, usually opposing, views. There may be an opportunity to develop more support for meat reduction policies within the population, if opinions are still being formed with regard to these policy suggestions, which may be relative newcomers to the public consciousness. Indeed, following the opportunity to consider such policies thoroughly, the UK Citizens Assembly on Climate Change expressed much higher levels of support for measures to reduce red meat consumption (between 60 and 80%) (Climate Assembly UK, 2020).

Strengths of this study include the large sample, using quotas representative of the UK population by gender, age and education. The study assessed a broader range of policies that could be used to target meat reductions than in previous research. In addition, by using a similar scale and study design to studies that assessed other policy targets we are able to compare and contrast levels of public support between these behavioural policy targets. The limitations of the study include the policies presented representing a relatively narrow range of the potential options that could be implemented and with just one example for each policy type, e.g. a 20% tax. Moreover, these policies were not described alongside the (best-available) evidence of their effectiveness, which could increase acceptability for those meat reduction policies that are less popular but potentially more effective (Reynolds, Stautz, Pilling, van der Linden, & Marteau, 2020). Finally, the framing manipulation may not have been sufficiently salient to participants, and the impact of pre-tested and/or enhanced framing could be explored in further work.

In conclusion, meat reduction policies attract less public support than those targeting reduced consumption of other foods and alcohol. Consistent with previous evidence, interventions relying on providing information had greater public support than interventions targeting industry actions or price. Less than half the population expressed specific support for individual policy actions to reduce meat consumption, regardless of framing measures as benefitting health or the environment. Nonetheless most of the policies assessed in the study (with the exceptions of banning advertising and price increases) were supported more than opposed, and many respondents were undecided, suggesting room for substantial segments of the population to potentially accept measures to reduce meat consumption. Further research is needed to identify interventions that could boost public support for meat reduction to encourage stronger policy action.

## Data sharing

The dataset generated during the current study is available in the Open Science Framework https://osf.io/gbq8p/.

## Author contributions

All authors contributed to the concept and design of the studies. RP developed the study on the survey platform, collected and analysed the study data and drafted the manuscript. All authors discussed the results and implications and commented on the manuscript.

## Funding

This research was funded by the 10.13039/100010269Wellcome Trust, Our Planet Our Health (Livestock, Environment and People: LEAP; award number 205212/Z/16/Z). TMM holds a Collaborative Award in Science from 10.13039/100010269Wellcome Trust (Behaviour Change by Design: 206853/Z/17/Z). The funder had no role in the study design, data collection, analysis, or interpretation. For the purpose of Open Access, the author has applied a CC BY public copyright licence to any Author Accepted Manuscript version arising from this submission.

## Declaration of competing interest

None
