# Supplementary material for: Acceptability of policies to reduce consumption of red and processed meat: A population-based survey experiment
Source: J Environ Psychol. 2022 Jun;81:101817. doi: 10.1016/j.jenvp.2022.101817 (PMC9742849; doi:10.1016/j.jenvp.2022.101817)
Supplement: Multimedia component 1 [file mmc1.docx]

**Acceptability of policies to reduce consumption of red and processed meat: A population-based survey experiment**

**Supplementary Materials**

**Table S1**. Regression analyses modelling mean policy acceptability by participant characteristics, run for the health and environment conditions separately.

**Table S2**. Regression analyses modelling mean policy acceptability by participant characteristics, without the intentions to reduce meat questions.

**Figure S1**. Support for policies by condition (from 0 “Strongly oppose” to 6 “Strongly support”)

**Table S1**. Comparing policy acceptability ratings between health and environment conditions

*NB: An alpha value of 0.008 (using a Bonferroni adjustment) was used for the difference tests*

|  | **Environment Framing Mean (s.d.)** | **Health**  **Framing Mean (s.d.)** | **Difference test*** | **Total Mean (s.d.)** | **Multilevel linear regression model** | | |
| --- | --- | --- | --- | --- | --- | --- | --- |
|  |  |  |  |  | **Coeff-icient** | **95% CIs** | **p-value** |
| Media campaign | 3.26 (1.77) | 3.22 (1.75) | Z=0.754, p=0.45 | 3.24 (1.76) | 1.01 | 0.95, 1.08 | <0.001 |
| Incentives for farmers | 3.13 (1.70) | 3.09 (1.66) | Z=0.719, p=0.47 | 3.11 (1.68) | 0.89 | 0.82, 0.95 | <0.001 |
| Labels | 3.39 (1.72) | 3.35 (1.69) | Z=0.763, p=0.45 | 3.37 (1.71) | 1.14 | 1.08, 1.21 | <0.001 |
| Reduced availability | 3.11 (1.78) | 3.07 (1.79) | Z=0.680, p=0.50 | 3.09 (1.78) | 0.87 | 0.81, 0.93 | <0.001 |
| Advertising ban | 2.64 (1.77) | 2.51 (1.75) | Z=1.929, p=0.05 | 2.58 (1.77) | 0.35 | 0.29, 0.42 | <0.001 |
| Increased price | 2.25 (1.92) | 2.20 (1.91) | Z=0.653, p=0.51 | 2.22 (1.91) | *Ref* |  |  |
|  | | | | | | | |
| Health frame |  |  |  | 2.91 (1.47) | -0.06 | -0.18, 0.07 | 0.371 |
| Environment frame |  |  |  | 2.96 (1.49) | *Ref* |  |  |

* Mann-Whitney tests comparing acceptability of health vs. environment conditions for individual meat reduction policies

**Table S2**. Regression analyses modelling mean policy acceptability by participant characteristics, run for the health and environment conditions separately.

*A p-value of <0.003 indicates significance*

|  | | **Environment** | | | **Health** | | |
| --- | --- | --- | --- | --- | --- | --- | --- |
|  |  | Coeff-icient | 95% CIs | p-value | Coeff-icient | 95% CIs | p-value |
| Age | | -0.003 | -0.006, 0.001 | 0.213 | -0.004 | -0.008, -0.000 | 0.041 |
| Gender  (ref: Male) | Female | -0.00 | -0.12, 0.12 | 0.999 | -0.04 | -0.15, 0.08 | 0.519 |
|  | Other | -0.79 | -1.90, 0.32 | 0.162 | 0.73 | -1.04, 2.50 | 0.420 |
| Education  (ref: Lower) | Higher | 0.04 | -0.08, 0.16 | 0.536 | 0.103 | -0.08, 0.15 | 0.571 |
| Ethnicity  (ref: Other) | White | -0.01 | -0.19, 0.18 | 0.935 | 0.05 | -0.13, 0.22 | 0.599 |
| Meat (any) consumption | | -0.01 | -0.05, 0.03 | 0.537 | **-0.08** | **-0.11, -0.04** | **<0.001** |
| Meat (red) consumption | | -0.04 | -0.08, -0.01 | 0.027 | -0.03 | -0.06, 0.00 | 0.072 |
| Intend to reduce meat consumption (any meat) | | **0.12** | **0.06, 0.19** | **<0.001** | 0.05 | -0.01, 0.11 | 0.092 |
| Intend to reduce meat consumption (red meat) | | **0.15** | **0.09, 0.21** | **<0.001** | **0.17** | **0.12, 0.23** | **<0.001** |
| Concern over population health | | 0.02 | -0.03, 0.08 | 0.376 | -0.03 | -0.08, 0.02 | 0.314 |
| Concern over environment | | **0.10** | **0.05, 0.15** | **<0.001** | **0.13** | **0.08, 0.18** | **<0.001** |
| Belief that eating less meat is good for health | | **0.15** | **0.09, 0.21** | **<0.001** | **0.16** | **0.10, 0.22** | **<0.001** |
| Belief that eating less meat is good for environment | | **0.22** | **0.16, 0.28** | **<0.001** | **0.22** | **0.16, 0.28** | **<0.001** |
| Trust in government | | **0.07** | **0.03, 0.11** | **<0.001** | **0.05** | **0.02, 0.09** | **0.003** |
| Constant | | 0.46 | 0.09, 0.84 | 0.016 | 1.04 | 0.69, 1.40 | <0.001 |

*N.B. 1052 observations (9 participants did not report ethnicity, 1 did not report either ethnicity or gender, 47 did not answer the intentions to reduce meat question (of whom 3 did not report ethnicity))*

*1045 observations for health analyses (7 participants did not report ethnicity, 1 did not report gender, 56 did not answer the intentions to reduce meat question)*

**Table S3**. Regression analyses modelling mean policy acceptability by participant characteristics, without the intentions to reduce meat questions.

*A p-value of <0.003 indicates significance*

|  | | **Coefficient** | **95% CIs** | **p-value** |
| --- | --- | --- | --- | --- |
| Age | | **-0.007** | **-0.010, -0.004** | **<0.001** |
| Gender (ref: Male) | Female | -0.01 | -0.10, 0.08 | 0.822 |
|  | Other | 0.01 | -0.86, 0.89 | 0.975 |
| Education (ref: Lower) | Higher | 0.05 | -0.04, 0.13 | 0.271 |
| Ethnicity (ref: Other) | White | 0.03 | -0.11, 0.16 | 0.685 |
| Meat (any) consumption | | **-0.10** | **-0.13, -0.08** | **<0.001** |
| Meat (red) consumption | | -0.02 | -0.05, 0.01 | 0.125 |
| Concern over population health | | -0.02 | -0.06, 0.01 | 0.198 |
| Concern over environment | | **0.14** | **0.10, 0.17** | **<0.001** |
| Belief that eating less meat is good for health | | **0.24** | **0. 20, 0.29** | **<0.001** |
| Belief that eating less meat is good for environment | | **0.30** | **0.26, 0.34** | **<0.001** |
| Trust in government | | **0.08** | **0.05, 0.11** | **<0.001** |
| Constant | | 1.18 | 0.92, 1.44 | <0.001 |

*N.B. 2197 observations (17 participants did not report ethnicity, 2 did not report gender (1 of whom did not report either ethnicity or gender)*
